# Differentials in lifestyle practices and determinants among hypertensive adults from three geopolitical zones in Nigeria

**DOI:** 10.11604/pamj.2024.48.98.40776

**Published:** 2024-07-11

**Authors:** Mobolaji Modinat Salawu, Justice Enosetale Erakhaiwu, Eniola Adetola Bamgboye, Rabiu Ibrahim Jalo, Okechukwu Samuel Ogah, Oyediran Emmanuel Oyewole, Joshua Odunayo Akinyemi, Mahmoud Umar Sani, Ikeoluwapo Oyeneye Ajayi

**Affiliations:** 1 Department of Epidemiology and Medical Statistics, Faculty of Public Health, College of Medicine, University of Ibadan, Ibadan, Nigeria,; 2 Department of Community Medicine, Bayero University, Aminu Kano Teaching Hospital, Kano, Nigeria,; 3 Department of Internal Medicine, Faculty of Clinical Sciences, College of Medicine, University of Ibadan, Ibadan, Nigeria,; 4 Department of Health Promotion and Education, Faculty of Public Health, College of Medicine, University of Ibadan, Ibadan, Nigeria,; 5 Department of Internal Medicine, Bayero University, Aminu Kano Teaching Hospital, Kano, Nigeria

**Keywords:** Hypertension, lifestyle practices, adults

## Abstract

**Introduction:**

hypertension is a major public health problem globally. The occurrence has been associated with unhealthy lifestyles (such as high salt consumption, physical inactivity, excessive intake of alcohol and unhealthy diet), which are very critical for hypertension control. The study was conducted to assess the lifestyle practices and their determinants among adults with hypertension in Nigeria.

**Methods:**

data on 762 adults living with hypertension were extracted from a cross-sectional survey conducted across three States (Abia, Kano and Oyo States) in Nigeria. A semi-structured pre-tested, interviewer-administered questionnaire was used for data collection. Knowledge of lifestyle practices was categorized into good and poor at 25^th^ percentile cut-off point. Overall lifestyle practice was grouped into healthy and unhealthy practices. Healthy lifestyle practice was defined as score of four and above while unhealthy lifestyle practice was defined as score of three and below; in all the 7 specific domains of lifestyle practices assessed (maximum obtainable was 7). The cut off was chosen based on 90% sensitivity from the Receiver Operating Curve (ROC) distribution of the scores. Data was summarized using descriptive statistics, Chi-square test and binary logistic regression were used to explore associations and determine predictors of lifestyle practices. Level of significance was set at 5%.

**Results:**

the mean age of the respondents was 55.4±16.3 years. About one-quarter of the respondents (24.3%) had good knowledge of lifestyle practices. Overall, 11.8% of respondents were engaged in good lifestyle practices. Independent predictors of good lifestyle practices were earning monthly income of N30,000 and above [AOR=1.58; 95% CI (1.03-2.42)], being a farmer [AOR=1.09; 95% CI (0.55-2.18)] and artisan [AOR=1.50; 95% CI (0.70-3.14)].

**Conclusion:**

the poor knowledge of lifestyle practices and engagement among adults with hypertension found in this study underscore the need to emphasize integrating lifestyle education for effective management of hypertension.

## Introduction

Globally, Hypertension (HTN) is a major public health problem affecting 1.28 billion people of which two-thirds resides in low-middle income countries [1]. It is the leading global cause of disability and premature mortality across all age groups [1,2]. In sub-Saharan Africa, hypertension is the most prevalent Cardiovascular Disease (CVD), affecting more than 20 million people accounting for major cardiovascular cause of hospitalization and death [3]. In Nigeria, hypertension and its complications are the most common non-communicable disease with a prevalence ranging from 8% to 32.5% in rural and urban communities respectively [4-7]. Hypertension is defined as the persistent elevation of the systolic and or diastolic blood pressure that is equal to or more than 140 and 90 mmHg respectively in adults aged 18 years and above [1,6,8]. Hypertension is a silent killer and can result in an increased risk of damage to the heart, kidney, brain and other diseases [9]. It may have no warning signs and symptoms thus, the diagnosis in many people is often an incidental finding when seeking treatment for unrelated ailments. In addition, if untreated, it may result in various complications such as coronary heart disease, stroke, congestive heart failure, renal insufficiency, peripheral vascular disease and ultimately death [10,11].

In a developing country like Nigeria, the high prevalence of HTN has been associated with urbanization and unhealthy lifestyle practices such as high salt consumption, physical inactivity, excessive intake of alcohol, tobacco use and unhealthy diet [12-15]. Tobacco use increases the risk of complications among persons with hypertension, causing approximately 6 million deaths yearly. This prevalence is expected to increase beyond 8 million deaths in the year 2030 if hypertension is left uncontrolled [16]. Alcohol consumption accounts for 5.9% of global mortality while unhealthy diets cause approximately 1.7 million deaths yearly [17,18]. Patients who have hypertension are expected to adhere to antihypertensive medication as well as other non-pharmacological measures in order to alleviate the burden imposed by the disease. Individuals with hypertension or pre-hypertension are advised to make lifestyle changes according to the 8^th^ Report of the Joint National Committee on Prevention, Detection, Evaluation, and Treatment of High Blood Pressure (JNC-8), WHO-ISH, and the Nigerian Hypertension Society (NHS) [19,20]. Non-pharmacologic therapy, currently known as lifestyle practice is defined as behavioral daily choices and practices by adults with hypertension as regards physical activity, alcohol consumption, tobacco consumption, fruits intake, vegetables intake, saturated fats and oils intake and high salt consumption [21]. This serves as an adjunct therapy for hypertension and include weight reduction, cessation of smoking, increased physical activity, consuming alcohol and dietary sodium in moderation and adhering to the Dietary Approach to Stop Hypertension (DASH) eating plan.

Observational epidemiological studies and randomized controlled trials have identified a dose-response association between dietary sodium intake, diet, exercise and blood pressure control in human population [22,23]. A study in Ghana reported that lifestyle practices such as increased physical activity, abstaining from alcohol and smoking, increased intake of fruits and vegetables, and reduced intake of carbohydrates, meat, and fat have a positive influence on blood pressure control [24]. Further, a clinical trial demonstrated the long term effects of weight loss and dietary sodium reduction on reduction in systolic blood pressure of 5mmHg and reductions of 14% in stroke mortality, 9% in mortality caused by heart disease, and 7% in all-cause mortality among participants in the intervention group [25]. Despite the known benefits of healthy lifestyle practices, there is poor knowledge and practice of healthy lifestyle among adult with hypertension patients in Nigeria which has contributed to the poor control of hypertension [26]. The challenge with management of hypertension especially non-adherence to healthy lifestyle practices cuts across the different parts of Nigeria. However, there are differences in culture and beliefs which may influence the practice of healthy lifestyle. To understand the possible differences, this study was conducted to assess the lifestyle practices among adults with hypertension in selected States representing three geopolitical zones and major ethnic groups in Nigeria. In addition, we investigated the factors associated with healthy lifestyle practices.

## Methods

### Study area

The study was conducted in three States of Nigeria namely, Abia, Kano and Oyo States. Abia State was created in 1991, located in the south eastern geopolitical zone of Nigeria and the capital is Umuahia. The major ethnic group of the State is the Igbo and the local language is Ibo. Kano State was created in 1968 and it's situated in the north-eastern geopolitical zone of Nigeria. The State is the second largest city in the nation after Lagos. The population of Kano State is made up mainly by Hausa and Fulani. Oyo State was created in 1976, it is located in south-western geopolitical zone of Nigeria and Ibadan is the capital. It is mainly inhabited by the Yoruba ethnic group. The data reported in this study were collected as part of the baseline assessment for a cluster-randomized trial on hypertension control among adult Nigerians. The study was conducted based on the published protocol on hypertension control in Nigeria [27].

### Study population

In this paper, the study sample comprised 762 adults with hypertension aged 18 years and above. The criteria for inclusion included old and newly diagnosed people living with hypertension, resident in the study sites for one year or more. Patients who were critically ill, or had mental disorder and pregnant women were excluded.

### Study design and data collection

The data analyzed for this paper were collected in the baseline survey for a larger study on development of a package to improve hypertension control among adult Nigerian. The baseline study was a cross-sectional design. The full description of the study design is available in the published protocol paper [27].

A semi-structured, pretested interviewer-administered questionnaire adopted from WHO STEPwise questionnaire was used for data collection. The questionnaire was translated to and administered in English, Yoruba, Igbo and Hausa languages depending on the study site. The questionnaire consists of four sections viz, sociodemographic characteristics, knowledge on lifestyle practices. Behavioral risk factor assessment and record of blood pressure and anthropometric measurements of the participants. Lifestyle practice was defined as behavioral daily choices and practices by adults with hypertension as regards physical activity, alcohol consumption, tobacco consumption, fruits intake, vegetables intake, saturated fats and oils intake and high salt consumption. Respondents' weight was measured using weighting scale and height was measured with use of pedometer.

A digital blood pressure monitor (Omron®) with appropriate cuff size was used to measure blood pressure. Participants rested for at least 10 minutes before measurement was taken. They sat quietly on a chair with feet flat on the floor and arm resting on a table which was approximately at the heart level. Two measurements were taken at least 2 minutes interval in the left arm while the participants were told to be calm and not talk as their Blood Pressure (BP) was being measured. The mean of the two readings was determined. Hypertension was defined as Systolic Blood Pressure (SBP)= 140 mmHg and/or Diastolic Blood Pressure (DBP)= 90 mmHg [1].

### Operational definitions

**Lifestyle modification:** also known as non-pharmacological therapy, which is the cornerstone of helping out patients with hypertension to attain lifestyle behaviours that are healthy including reduction of salt, regular exercise, reduction of alcohol, avoiding smoking, increasing fruit and vegetable and others. They are [28,29]: 1) reduction of salt is consuming less than 5 grams (2 teaspoons) of salt per day. 2) Regular exercise is performing moderate-intensity exercises for at least 150 minutes a week or 75 minutes of vigorous-intensity exercises a week which could be aerobic physical activity or some equivalent combinations. 3) Reduction of alcohol consumption is beverages intake limited to 2 drinks per day (20 g/d of alcohol) for men and one drink per day (10 g/d of alcohol) for women. 4) Smokers on an active act of smoking a particulate cigarette. 5) Improving diet and nutrition such as increasing fruit and vegetable consumption is taking greater than or equal to 400 grams per day (2-3 serving for each), and reduction of saturated fats intake.

Body Mass Index (BMI) was calculated using weight in kilogram (kg) divided by square of height in meters (m). It was classified using WHO classification of BMI into: underweight (<18.5), normal weight (18.5 - 24.9), overweight (25.0 - 29.9), obese (>30.0) [30].

### Variables

**Independent variables:** the independent or explanatory variables consist of respondents' sociodemographic information, such as age, sex, level of education, marital status, monthly income, location, religion, ethnicity, occupation, and States.

### Outcome measures

Respondents' knowledge of lifestyle practices was assessed based on 15 item-questions. Incorrect response to questions on knowledge of lifestyle practices was scored “0”, while correct response was scored “1”. Knowledge score among the respondents was not normally distributed, hence percentiles was used to classify the score into poor (25^th^ percentile), fair (>25^th^ < 75^th^ percentile) and good (= 75^th^ percentile). Those who scored 0-6 were judged to have poor knowledge; 7-11 had fair knowledge while 12-15 had good knowledge of lifestyle practices.

Respondents' lifestyle practices were assessed in seven domains were assessed. Each domain of lifestyle practice was given an ordinal score as follows: physical activity practice= 1 point, non-alcohol consumption= 1 point, non-use of tobacco= 1 point, dietary salt intake reduction = 1 point, fruits consumption= 1 point, vegetables consumption= 1 point and use of dietary fats and oils= 1 point. Physical activity was assessed based on WHO recommendation (at least 150 minutes moderate physical activity and at least 75 minutes of vigorous physical activity per week). Respondents who met this recommendation were classified as being physically active. Non-alcohol and tobacco consumption were based on 100% abstinence by respondents. Dietary salt intake practice was based on non-addition of raw table salt in addition to the one used to prepare the food item during meal times. Fruits and vegetables consumption by respondents were based on its intake at least once in a week. The fats and oils were categorized into saturated and unsaturated fats and oils based on the type of oils and fats available in Nigeria. Respondents practice of healthy dietary fat and oil consumption was based on unsaturated fats and oils consumption. Overall lifestyle practice was grouped into healthy and unhealthy practices. Healthy lifestyle practice was defined as score of four and above while unhealthy lifestyle practice was defined as score of three and below, in all the 7 specific domains of lifestyle practices assessed (maximum obtainable was 7).

### Data analysis

Data were analyzed using statistical software package (Stata version 16). Data summarization was done using descriptive statistics such as mean, frequency, and proportion. Inferential statistics such as Chi-square test was used to test the association between lifestyle practices and explanatory variables including sociodemographic and knowledge of lifestyle practice. Variables significant at 10% were introduced into the binary logistics regression model. Determinants of healthy lifestyle practices in each State were assessed using binary logistic regression analysis. Level of significance was set at p<0.05.

## Results

### Sociodemographic characteristics

Table 1 shows the sociodemographic information of the respondents. The mean (SD) age of respondents was 55.4 (16.32) years. Majority 350 (45.9%) of the respondents were = 60 years of age while the least, 131 (17.2%), were < 40 years of age. Six hundred and fifteen (80.7%) were married. Two-thirds 431 (65.5%) of the respondents earned below minimum wage of N30,000 (60 USD) per month. Female constituted higher proportion of the study respondents 480 (62.9%). The three study states were all represented in the population: Abia 304 (39.9%), Kano 283 (37.1%) and Oyo 175 (22.9%).

**Table 1:** sociodemographic of respondents

Socio-demographic characteristics	Frequency	Percentage
**Age group (years)**		
<40	131	17.2
40-59	128	36.9
≥60	350	45.9
Sex		
Male	282	37.0
Female	480	62.9
**Marital Status**		
Currently Married	615	80.7
Divorced/Separated/Widowed	114	15.0
Never Married	33	4.3
**Education**		
No formal education	258	33.9
Primary	197	25.9
Secondary	192	25.2
Tertiary	115	15.1
**Monthly income**		
<30,000	431	65.5
≥30,000	227	34.5
Location		
Urban	396	52.0
Rural	366	48.0
**Religion**		
Christianity	387	50.8
Islam	372	48.9
Traditional	3	0.4
**Ethnicity**		
Igbo	315	41.3
Hausa	282	37.0
Yoruba	165	21.7
**Occupation**		
Petty trade/Business	235	30.8
Farming	180	23.6
Unemployed	126	16.5
Civil servant	81	10.6
Artisanry	76	10.0
Retired	64	8.4
**State**		
Abia	304	39.9
Kano	283	37.14
Oyo	175	22.97

### Knowledge of lifestyle practices among people living with hypertension

The knowledge on lifestyle practices was found to be good among 24.3% of the respondents while, 35.3% had fair and (40.4%) had poor knowledge.

### Lifestyle practices by specific domains among people living with hypertension

Table 2 shows the distribution of the patients based on specific domain of lifestyle showed that 26 (8.6%), 53 (7.0%) and 18 (10.3%) respondents have ever smoked in Abia, Kano and Oyo States respectively. Alcohol consumption in the past 30 days prior to the study was practiced by 69 (65.7%), and 29 (45.3%) in Abia and Oyo States respectively. None of the respondents in Kano reported alcohol consumption. Most respondents consumed vegetables at least once a week across the three States Abia 263 (86.8%), Kano 238 (84.1) and Oyo 145 (82.9). About half of respondents in Abia State practiced physical activity 167 (54.9), followed by Oyo State 79 (45.1%) and Kano 80 (28.3%).

**Table 2:** practice of specific domain of lifestyle among people living with hypertension by States

Variable	Abia	Kano	Oyo
**Ever smoked**			
No	227(91.4)	274(96.8)	157(89.7)
Yes	26(8.6)	53(7.0)	18(10.3)
**Currently smoke**			
No	19(73.1)	-	9(50.0)
Yes	7(26.9)	-	9(50.0)
**Ever taken alcohol**			
No	200(65.8)	282(99.7)	111(63.4)
Yes	104(34.2)	1(0.4)	64(36.6)
**Consumed alcohol in the past 30 days**			
No	36(34.3)	1(100.0)	35(54.7)
Yes	69(65.7)	-	29(45.3)
**Add salt to prepared food**			
No	255(83.9)	250(88.3)	155(88.6)
Yes	49(16.1)	33(11.7)	20(11.4)
**Dietary fat/oil consumption**			
No	1(0.3)	44(15.6)	1(0.6)
Yes	303(99.7)	239(84.5)	174(99.4)
**Frequency of fruit consumption**			
Daily	30(9.9)	12(4.2)	24(13.7)
At least once a week	263(86.8)	238(84.1)	145(82.9)
Never	10(3.3)	33(11.7)	6(3.4)
**Frequency of vegetable consumption**			
Daily	24(7.9)	38(13.4)	13(7.4)
At least once a week	280(92.1)	245(86.6)	162(92.6)
**Physically active**			
No	137(45.1)	203(71.7)	96(54.9)
Yes	167(54.9)	80(28.3)	79(45.1)

Overall assessment of lifestyle practices among the respondents showed that 144 (18.9%) had healthy lifestyle practices whilst 618 (81.1%) had unhealthy lifestyle practices.

### Factors associated with lifestyle practices among total population of people living with hypertension

At bivariate analysis, factors significantly associated with healthy lifestyle practices among the total population of adults with hypertension were: monthly income (Χ^2^= 5.53, p-value=0.019), and occupation (Χ^2^2=20.73, p-value = 0.001) while other factors were not statistically significant. The results showed that 23.8% of people living with hypertension with monthly income above N30,000 compared to 16.2% in those with income below N30,000 had healthy lifestyle practices. In terms of occupation, artisans had the highest proportion with healthy lifestyles (Table 3).

**Table 3:** factors associated with respondents' practices

Socio-demographic characteristics	Life>		Chi-square	p-value
	Unhealthy	Healthy		
**Age-group years**				
<40	105(80.2)	26(19.9)	5.58	0.061
40-59	217(77.2)	64(22.8)		
≥60	296(84.6)	54(15.4)		
**Sex**				
Male	233(82.6)	49(17.4)	0.67	0.411
Female	385(80.2)	95(19.8)		
**Marital status**				
Currently married	499(81.1)	116(18.9)	0.75	0.687
Never married	25(75.8)	8(24.2)		
Divorced/separated/widowed	94(82.5)	20(17.5)		
**Education**				
No formal education	218(84.5)	40(15.5)	4.649	0.199
Primary	155(78.7)	42(21.3)		
Secondary	149(77.6)	3(22.4)		
Tertiary	96(83.5)	19(16.5)		
**Monthly income**				
<30,000	361(83.8)	70(16.2)	5.537	0.019*
≥30,000	173(76.2)	54(23.8)		
**Location**				
Rural	302(82.5)	64(17.5)	0.915	0.339
Urban	316(79.8)	80(20.2)		
**Religion**				
Christianity	306(79.0)	81(20.9)	2.659	0.265
Islam	310(83.3)	62(16.7)		
Traditional	2(66.7)	1(33.3)		
**Ethnicity**				
Hausa	237(84.0)	45(16.0)	2.758	0.252
Igbo	252(80.0)	63(20.0)		
Yoruba	129(78.2)	36(21.8)		
**Occupation**				
Civil servant/professionals	63(77.8)	18(22.2)	20.734	0.001*
Farming	140(77.8)	40(22.2)		
Artisanry	55(72.4)	21(27.6)		
Trading	186(79.2)	49(20.9)		
Unemployed	113(89.7)	13(10.3)		
Retired	61(95.3)	3(4.7)		
**BMI**				
Underweight	33(75.0)	11(25.0)	4.165	0.244
Normal	281(81.9)	62(18.1)		
Overweight	158(84.5)	29(15.5)		
Obese	145(77.5)	42(22.5)		
**Knowledge of life>**				
Poor	244(79.2)	64(20.8)		
Fair Good	230(85.5) 144(77.8)	39(14.5) 41(22.2)	5.395	0.067

Significant variable*

### Predictors of healthy lifestyle practices among total population of people living with hypertension

Table 4 shows the unadjusted odds of healthy lifestyle among the total population. The significant predictors of healthy lifestyles were age, sex, monthly income, and ethnicity. Respondents aged 40-59 years [OR=1.76, 95% CI (1.04-2.96)] and 60 years and above [OR= 2.06, 95%CI (1.14-3.71)] were more likely to practice healthy lifestyle compared to those less than 40 years of age. Females [OR= 2.23, 95% CI (1.44-3.46)] were twice more likely to practice healthy lifestyle than males. Respondents with monthly income above 30,000 (60 USD) [OR=1.58, 95% CI (1.05-2.38) were more likely to practice healthy lifestyle. According to the ethnicity, Igbo [OR=0.37, 95% CI (0.22-0.63)] and Yorubas [OR=0.40, 95% CI (0.23-0.70)] were less likely to practice healthy lifestyle compared to the Hausas. People living with hypertension who were farmers [OR1.15= 95% CI (0.56-2.37)] and artisans [OR=1.08, 95% CI (0.50-2.34)] had higher odds of practicing healthy lifestyle compared to those in civil service.

**Table 4:** overall predictors of healthy lifestyle practice among the total population

Selected variables	OR	95%CI	p-value
**Age group**			
Less than 40 years	1+		
40-59 years	1.76	1.04-2.96	0.03
60 years and above	2.06	1.14-3.71	0.01*
**Sex**			
Male	1+		
Female	2.23	1.44-3.46	0.001*
**Marital status**			
Married	1+		
Never married	1.68	0.63-4.46	0.29
Divorced/separated	1.64	0.90-2.98	0.10
**Education**			
No formal	1+		
Primary	1.22	0.72-2.05	0.44
Secondary	1.41	0.80-2.49	0.23
Tertiary	1.15	0.59-2.27	0.66
**Monthly income**			
Less than N30,000	1+		
Above N30,000	1.58	1.05-2.38	0.02
**Location**			
Rural	1+		
Urban	1.28	0.88-1.88	0.19
**Ethnicity**			
Hausa	1+		
Igbo	0.37	0.22-0.63	0.001
Yoruba	0.40	0.23-0.70	0.001
**Occupation**			
Civil servant	1+		
Farming	1.15	0.56-2.37	0.68
Artisan	1.08	0.50-2.34	0.83
Trading	0.94	0.48-1.85	0.87
Unemployed	0.38	0.16-0.88	0.02
Retired	0.68	0.29-1.58	0.37

Statistical significance * Reference category +

### Predictors of healthy lifestyle practices among people living with hypertension by States

The unadjusted odds of healthy lifestyle among the respondents in each of the State is presented thus. In Abia State the association between monthly income above 30,000 (60 USD) and health lifestyle practices was sustained (OR=3.00, CI: 1.24, 7.25). For Kano State, age group and occupation were the only significant factors. People living with hypertension aged 40-59 years were more likely of health lifestyle practices compared to those aged less than 40 years. The unemployed people living with hypertension in Kano were less likely to practice healthy lifestyles compared to civil servants (OR=0.19, CI: 0.04, 0.84). In Oyo State, the odds of healthy lifestyle practice increased with age of people living with hypertension: age 40-59 (OR=4.16, CI: 1.30-13.2); age > 60 years (OR=10.2, CI: 2.39-43.6). Women were almost 8 times as likely as men to practice healthy lifestyle (OR = 7.77, CI: 2.73, 22.0) (Table 5).

**Table 5:** predictors of healthy lifestyle practice among people living with hypertension by States

	Abia State			Kano State			Oyo State		
Selected variables	OR	p-value	95%CI	OR	p-value	95%CI	OR	p-value	95%CI
**Age group (years)**									
Less than 40	1+			1+			1+		
40-59	0.88	0.81	0.31-2.50	2.26	0.04	1.00-5.07	4.16	0.01	1.30-13.2
60 and above	0.74	0.60	0.24-2.29	2.57	0.06	0.95-6.93	10.2	0.00	2.39-43.6
**Sex**									
Male	1+			1+			1+		
Female	1.95	0.05	0.98-3.90	1.69	0.32	0.58-4.88	7.77	0.00	2.73-22.0
**Marital status**									
Married	1+			1+			1+		
Never Married	1.87	0.50	0.29-11.7	1.82	0.54	0.25-13.1	1.71	0.56	0.27-10.8
Divorced/separated	4.40	0.05	0.94-20.4	1.00	0.98	0.34-2.94	1.31	0.52	0.47-3.67
**Education**									
No formal	1+			1+			1+		
Primary	0.89	0.83	0.30-2.61	1.10	0.83	0.45-2.69	2.39	0.14	0.74-7.64
Secondary	0.95	0.93	0.29-3.11	1.20	0.70	0.46-3.10	5.30	0.01	1.47-19.0
Tertiary	0.60	0.45	0.16-2.24	1.10	0.86	0.34-3.58	4.94	0.06	0.93-26.1
**Monthly income**									
Less than N30,000	1+			1+			1+		
Above N30,000	3.00	0.01	1.24-7.25	1.48	0.26	0.74-2.99	0.98	0.97	0.43-2.21
**Location**									
Rural	1+			1+			1+		
Urban	1.29	0.40	0.70-2.38	0.98	0.96	0.51-1.90	2.18	0.12	0.80-5.89
**Occupation**									
Civil Servant	1+			1+			1+		
Farming	2.79	0.11	0.76-10.1	0.48	0.27	0.13-1.76	3.35	0.25	0.41-27.0
Artisan	1.68	0.45	0.42-6.64	0.42	0.27	0.09-1.96	3.76	0.09	0.79-17.7
Trading	2.14	0.23	0.64-7.10	0.50	0.33	0.12-2.02	1.43	0.63	0.32-6.26
Unemployed	6.99	0.13	0.56-87.4	0.19	0.02	0.04-0.84	0.49	0.56	0.04-5.32
Retired	1.78	0.43	0.42-7.60	0.26	0.09	0.05-1.23	1.66	0.63	0.21-13.1

Reference category +

## Discussion

Healthy lifestyle practices play an essential function in the control of hypertension as well as in minimizing its complications. This study was conducted to assess the lifestyle practices and their determinants among adults with hypertension in three States of Nigeria (Abia, Kano and Oyo States) representing the three major ethnic groups with different culture and variety of food which may influence lifestyle practices.

In this study, only one-quarter of the participants had good knowledge of lifestyle practices, fair knowledge was reported among over a quarter while almost half of the respondents had poor knowledge of lifestyle practices. Our finding is similar to other community-based studies conducted in the south east, south west and northern part of Nigeria which reported inadequate knowledge of healthy lifestyle practices. The studies documented lack of awareness that regular exercise, moderation of alcohol intake, adequate consumption of vegetables, fruits and unsaturated oil are part of lifestyle practices for hypertension control [26,31]. Studies from other African countries such as Ghana, Ethiopia and Asian countries such as Karachi also demonstrated poor awareness of lifestyle practices for hypertension control [24,32,33]. However, in another study in East Africa, a high knowledge towards lifestyle practices was reported among patients with hypertension. This was said to be one of the rare findings in low-income countries considering the poor access to medical care and appropriate information [34]. Variations in the findings might be as a result of lack of healthcare workers with skill set for counselling on lifestyle practices for hypertension control.

In this study, majority of the respondents had unhealthy lifestyle practices. According to the ethnicity, Igbo and Yorubas were less likely to practice healthy lifestyle compared to the Hausas. This is possibly because the northerners which comprised of Hausas and Fulanis are known to consume more milk and nuts, known to be nutritious, and they use less cooking oil as compared with Igbo and Yoruba who consume cooking oil in larger quantity. High consumption of fatty foods is also common in the south among the Yorubas and Igbos. There was also variation in intake of vegetables and physical activity among respondents in the different States. This corroborates findings from previous studies in other parts of the country that reported inadequate lifestyle practices [31,35]. Majority of the respondents who were currently smoking and taking alcohol were residents of Abia and Oyo States. Low or no alcohol and smoking in Kano being a northern State might be based on religious principle. Studies from other Muslim dominated countries such as Iran, and several regions in Ethiopia, also documented similar findings [32,36,37]. This could also be due to the fact that various control strategies have been put in place to control tobacco intake in various parts of the world such as the use of print media, posters, billboards, radio and television adverts in rejecting tobacco use [14]. Policy implementation such as raising taxes on tobacco products and health talks on health effects of smoking has also demonstrated significant impact on reduction of tobacco smoking [14]. In another study by Findlow *et al*. among African Americans with hypertension, it was reported that weight management and salt reduction was a challenge [38]. However, healthy lifestyle practices were reported in studies conducted in India, Nepal and Ghana [36,39].

In our study, consumption of vegetables and fruits at least once a week featured prominently in the domains of lifestyle practices. Igbo respondents had higher rate of consumption of vegetables and fruits followed by the Hausa and then Yoruba respondents. Similar finding was reported in a study conducted on lifestyle practices among adults with hypertension in Abia State, Nigeria. This is a healthy behavior considering the high water, fiber and low-fat content of vegetables and the huge benefits on cardiovascular health [40]. However, daily consumption of vegetables and fruits was below ten percent across the States. This is low compared to a study among adults in Oyo State which reported a prevalence of 27% of daily vegetables and fruits intake [41]. Another study in Port Harcourt, Nigeria reported poor consumption of vegetables and fruits among their respondents [35]. In a study in low middle-income countries, low fruit and vegetable consumption prevalence ranged from 36.6% (Ghana) to 99.2% (Pakistan) for men and from 38.0% (Ghana) to 99.3% (Pakistan) for women. This was reported to be less than the minimum recommended five daily servings of fruits and vegetables recommended by WHO [42]. The reason for this could not be completely ascertained, however, we posit it may be as a result of low income and educational status of majority of respondents in the study which may limit their purchasing power.

Physical activity was the least lifestyle that the participants engaged in, and low practice/engagement was commonest among the respondents in Abia compared to Oyo and Kano. This is comparable to studies from eastern and northern Nigeria, Ethiopia, Israel and Bangladesh which also reported inadequate practice among the respondents [26,35,43-45]. This consolidates reports asserting that sedentary lifestyle is a health challenge in low- and middle-income countries causing an increase in the prevalence of chronic diseases like HTN and poor control. However, this is in contrast with the result from studies in Saudi Arabia and USA which reported much practice of physical activity [46, 47]. This discrepancy could be due to difference in individual level of awareness of lifestyle practices because of socio-economic status, unavailability of structures or material to engage in workout and other exercises.

Significant predictors of healthy lifestyle practice among the respondents were age, sex, monthly income and ethnicity. Respondents who were older than 40 years were more likely to practice healthy lifestyle compared to those less than 40 years. In a study by Cockerham *et al*. it was reported that age 40 plus is the age at which health problems linked to the cumulative effects of poor behaviors such as smoking, excessive alcohol intake, inadequate vegetables and fruit intake, poor dietary plan among others begin to surface. This is the age at which diseases such as obesity, alcoholism, high blood pressure, diabetes, high cholesterol, chronic inflammation, heart disease, and various other causes or contributors to mortality become more common [48]. Therefore, persons aged 40 years and above tend to be more interested in being proactive and adopting healthy lifestyle practices to stay in good health, avoid complications and death. However, for some people, they have always been protective and routinely living a healthy lifestyle.

Our findings showed that respondents with higher monthly income were more likely to practice healthy lifestyle. Cockerham *et al*. noted similar findings in a study among adults aged forty years and above that reported respondents who practiced healthy lifestyles have the highest incomes [48]. Individuals with low monthly income may not be able to acquire and maintain healthy diets on a regular basis. It is also said that healthy food does not come cheap compared with junk food [49]. They may also not be able to afford facilities and equipment that are conducive for physical activity such as the gym.

In addition, occupation played an important role in how participants engaged in lifestyle practices in this study. This is however, not statistically significant. Respondents who were unemployed were less likely to engage in lifestyle practices as compared to civil servants. Moreover, retired respondents were less likely to engage in lifestyle practices as compared to individuals who were engaged in one job or the other. Age and strength may also constitute a challenge for the retirees to engage in physical activity as a result of decline in bodily vitality with ageing process.

### Study limitations

The findings of this study are timely and generalizable as hypertension is on the rise among Nigerian adult population. This study is one of the few papers to report information on lifestyle practices and its differentials among adults with hypertension from multiethnic dimension, for the improvement of hypertension control in Nigeria. In addition, the use of WHO STEPwise questionnaire in data collection allowed for extensive retrieval of essential data. However, the following limitations were noted; the cross-sectional design of the study does not establish causality of the outcome and the ethnic diversity for settings outside the study country, may hinder generalizability of the study findings.

## Conclusion

Knowledge and practice of lifestyle practice among adults with hypertension in the three States studied were poor. The variation among the tribes showed that engagement in lifestyle practices is not completely based on ethnicity but, other factors such as sociodemographic characteristics of the individual play huge role. However, these variations in the domains of lifestyle practices observed among the three main ethnic groups in Nigeria were noticeable. Stakeholders in healthcare need to intensify efforts in educating adults on hypertension, the risk factors and lifestyle practices through an ethnic lens. This will help to mitigate the development of hypertension and ensure optimal control of the disease.

### What is known about this topic

- *Prevalence of hypertension among adults in Nigeria has been reported in many published primary research and systematic reviews to range from 30.6% to 38.1%;*
- *Risk factors for hypertension in adults are known in the epidemiology of hypertension in Nigeria;*
- *Overweight and obesity have been reported as important risk factors; others are, alcohol use, high blood sugar and cholesterol, physical in activity and family history of hypertension. Studies have also documented low fruits and vegetables consumption as risk factors for hypertension.*

### What this study adds

- *This is the first study to provide scientific evidence about ethnic differential on lifestyle practices of adults with hypertension in Nigeria as a singular study that showed ethnic variation in lifestyle practices among adults with hypertension in the Igbo and Yoruba ethnic groups were less likely to practice healthy lifestyle compared to the Hausas;*
- *Consumption of vegetables and fruits at least once a week featured prominently in the domains of lifestyle practices; respondents who were Igbo had higher rate of vegetables and fruits consumption compared to the Hausa and Yoruba respondents;*
- *Physical activity was the least lifestyle that the respondents engaged in; low engagement was the commonest among the respondents in Abia compared to Oyo and Kano.*
